# Rare cutaneous manifestation of COVID-19 infection and Pfizer-BioNTech COVID-19 vaccine with a unique pattern similarity

**DOI:** 10.2217/fvl-2021-0129

**Published:** 2021-10-19

**Authors:** Anthony Fata, Georges Jabbour, Hampig Kourie, Marianne Zoghbi, Elia Kassouf, Antoine Zoghbi

**Affiliations:** 1 ^1^Faculty of Medicine, Saint Joseph University of Beirut, Beirut, Lebanon; 2 ^2^Hotel-Dieu de France Hospital, Beirut, Lebanon

**Keywords:** COVID-19, cutaneous, dermatological, lesions, maculopapular, vaccine

## Abstract

In December 2019, a new emerging virus causing mild-to-severe pneumonia was detected in China. The virus was described as a variant of SARS-CoV and was called SARS-CoV-2, then declared a pandemic by the WHO on 11 March 2020. Millions of people contracted the virus and presented with a symptomatology of variable severity, including upper respiratory tract symptoms, systemic symptoms and diarrhea. We herein report a rare skin presentation in a 33-year-old female that occurred both during COVID-19 infection and after receiving the first dose of COVID-19 vaccine.

## Background

In December 2019, in the city of Wuhan, severe respiratory infections had been reported and seemed to develop into an acute respiratory distress syndrome (ARDS). Identified in January 2020 and labeled as a pandemic by the WHO in March 2020, the virus became a global health issue and caused a worldwide sanitary crisis [1]. COVID-19 is caused by the SARS-CoV-2 virus binding to type 2 alveolar cells in the lung with ACE 2 receptor by a surface spike protein. It can also interact with different cells in the GI tract, kidney, heart and endothelium causing multiorgan failure [2]. The ability of the virus to bind to different cells explains the broad range of symptoms that it can cause. Skin lesions have been reported as a possible symptom of COVID-19. The skin lesions related to this virus consist of five patterns: livedo or necrosis, maculopapular eruptions, urticarial lesions, vesicular eruptions and pseudo-chilblain lesions [3]. Here, we describe the case of a patient with an uncommon pattern of maculopapular eruption during SARS-CoV-2 infection and after receiving COVID-19 vaccine.

## Case report

We present the case of a 33-year-old female patient, working as a nurse in the medicine department. She was admitted to the emergency unit on 15 November 2020 for oppressive chest pain, fever, cough, tachycardia and fatigue. A PCR test was positive on 10 November with a cycle threshold (CT) of 18. The severe symptomatology started 24 h before presenting to the emergency department. She also noted having some chills and generalized fatigue within the previous 72 h. The patient was conscious and oriented in space, time and toward people. Blood pressure on admission was 140/80 mmHg, heart rate was 100 beats/min. Patient respiratory rate was 16 breaths/min and SPO_2_ was 94% on room oxygen. The patient had fever with a body temperature of 38.8°C.

The patient does not have any known or documented cardiovascular risk factor: no history of tobacco consumption, dyslipidemia, diabetes or cardiac problems. She is allergic to amoxicillin, with an allergic reaction of a cutaneous rash and angioedema. She also has an intolerance to butylscopolamine, resulting in agitation and irritability. She cannot take metoclopramide as it once resulted in hypotension.

A blood test performed on 15 November showed slightly increased C-reactive protein (CRP) and d-dimer levels. Full results are shown in Table 1.

**Table 1.** Results of blood analysis (hematology, biochemistry, immunology), 15 November 2020.

Blood analysis	Results	Normal value
White blood cells	4300/mm^3^	4000–10,000/mm^3^
Red blood cells	5.12 10^6^/mm^3^	4.3–5.7 10^6^/mm^3^
Hemoglobin	13.4 g/dl	14–17.5 g/dl
Hematocrit	40.3%	41.5–50.4%
MCV	79 fl	80–98 fl
MCHC	33.3 g/dl	32–36 g/dl
MCH	26.2 pg	27.5–33.2 pg
RDW	15.7%	10–15%
Platelets	175/mm^3^	150,000–400,000/mm^3^
Neutrophils (%)	71.4%	43–70%
Lymphocytes (%)	22.8%	38–42%
Monocytes (%)	5.3%	5–10%
Eosinophils (%)	0.2%	0–5%
Basophils (%)	0.3%	0–1%
CRP	6.5 mg/l	<3.5 mg/l
D dimer	0.77 μg/ml	<0.5 μg/ml
Ferritin	29 ng/ml	13–150 ng/ml
Creatinin	46 μmol/l	46–92 μmol/l

CRP: C-reactive protein; MCH: Mean corpuscular hemoglobin; MCHC: Mean corpuscular hemoglobin concentration; MCV: Mean corpuscular volume; RDW: Red cell distribution width.

**Figure 1.**
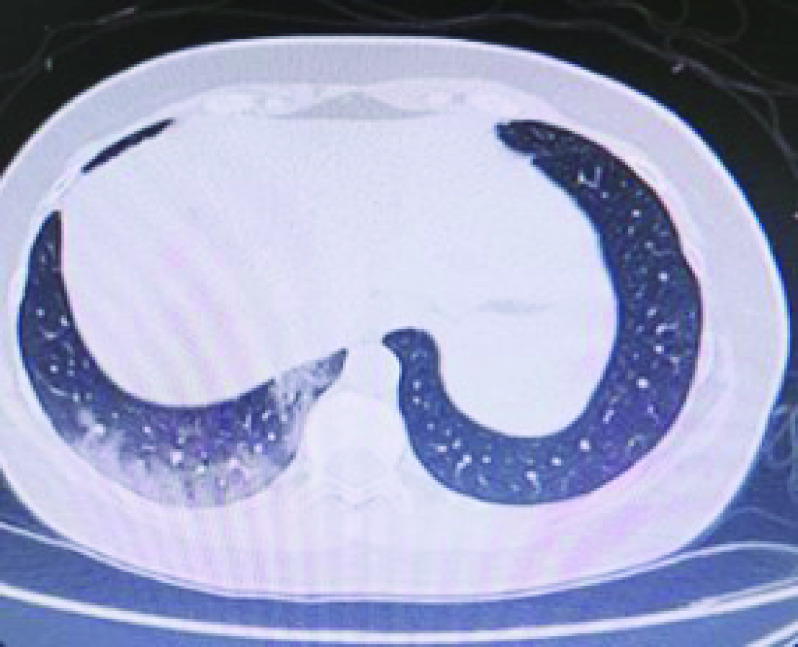
Thoracic computed tomography scan, 15 November 2020.

A thoracic computed tomography (CT) scan performed on 15 November 2020 showed right inferior lobar pneumonia and typical COVID-19 ground glass pattern (Figure 1). No adenomegaly was noted in the mediastinum and no pleural or pericardial effusion was found.

The patient did not require hospitalization due to the absence of comorbidities, a moderate lung inflammation on the scan and stable vital signs without a supplementary need of oxygen. She was released and a medical home treatment was started. It included oral corticosteroid therapy (deflazacort 15 mg per day for 10 days), anti-coagulation (fondaparinux 2.5 mg per day for 1 month), prophylactic antiplatelet dose (acetylsalicylic acid 100 mg per day for 2 months), an analgesic and antipyretic (paracetamol 1000 mg per 8 h) and supplements (vitamin C, vitamin D, zinc).

**Figure 2.**
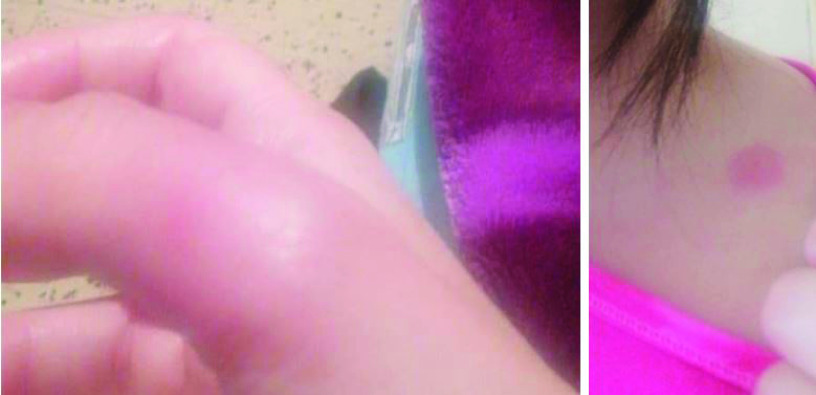
The evolution pattern of skin lesions, following COVID-19 infection, progressing from small vesicles into itchy and painful maculopapular lesions, then regressing before they disappear, 12 November 2020.

**Figure 3.**
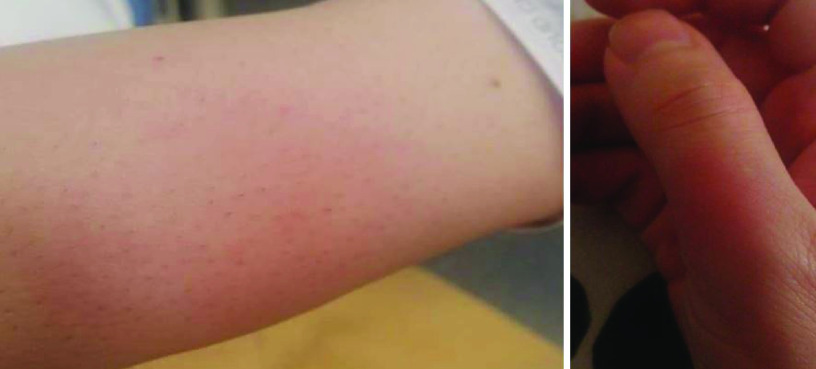
The evolution pattern of skin lesions, following COVID-19 infection, progressing from small vesicles into itchy and painful maculopapular lesions, then regressing before they disappear, 14 November 2020.

**Figure 4.**
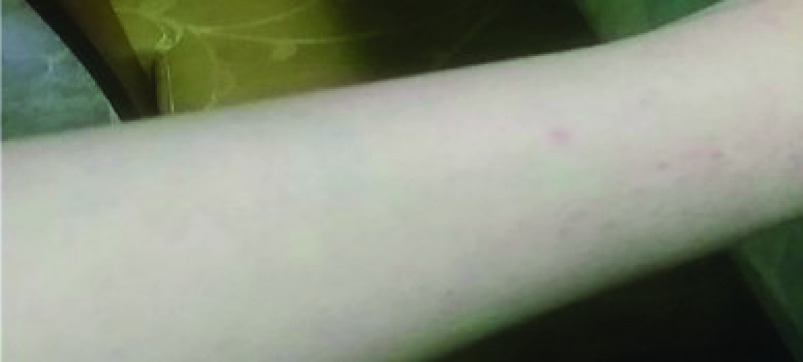
The evolution pattern of skin lesions, following COVID-19 infection, progressing from small vesicles into itchy and painful maculopapular lesions, then regressing before they disappear, 16 November 2020.

The onset of COVID-19 infection was on 10 November 2020, and involved the skin rather than respiratory system or general state. The skin lesions presented with an uncommon pattern starting with small vesicles on the forearms and the left sub clavicular area on 10 November 2020. On this day, a COVID-19 PCR test was positive with a CT value of 18. This was the first symptom of the infection and other symptoms occurred 24 h later. The skin vesicles evolved into itchy, painful, red, swollen maculopapular lesions (Figures 2 & 3) with progressive regression and complete resolution in one week (Figure 4).

After this atypical presentation, the patient gave us further information about her personal medical history, mentioning severe childhood viral infections, varicella infection despite vaccination (during childhood), mumps infection despite vaccination (during childhood), and severe H1N1 infection (in 2017). The patient had no history of personal or family auto-immune disease, no chronic or intermittent arthralgia or myalgia.

A complete blood analysis was done on 25 November 2020 and the results are shown in Table 2. CRP and d-dimer levels were normalized. Two PCR tests were done systematically on 24 and 27 November 2020, and were negative. The patient noted an improvement of COVID-19 symptoms. d-dimers were decreasing.

**Table 2.** Showing results of blood analysis (hematology, biochemistry, immunology), 25 November 2020.

Blood Analysis	Results	Normal value
White blood cells	9000/mm^3^	4000–10000/mm^3^
Red blood cells	5.21 10^6^/mm^3^	4.3–5.7 10^6^/mm^3^
Hemoglobin	13.9 g/dl	14–17.5 g/dl
Hematocrit	40.7%	41.5–50.4%
MCV	78 fl	80–98 fl
MCHC	34.1 g/dl	32–36 g/dl
MCH	26.6 pg	27.5–33.2 pg
RDW	15.8%	10–15%
Platelets	230/mm3	150000–400000/mm^3^
Neutrophils	62.8%	43–70%
Lymphocytes	29.7%	38–42%
Monocytes	5.9%	5–10%
Eosinophilis	1.4%	0–5%
Basophils	0.2%	0–1%
CRP	<3.12 ng/ml	0–9 ng/ml
Urea	3.5 mmol/L	2.5–7.5 mmol/L
Creatinin	46 μmol/L	46–92 μmol/L
Na	140 meq/L	135–145 meq/L
K	4.1 meq/L	3.5–4.5 meq/L
Cl	103 meq/L	98–107 meq/L
Alkaline reserve	27 meq/L	24–30 meq/L
d-dimers	0.4 *μ*g/ml	<0.5 *μg*/ml

CRP: C-reactive protein; MCH: Mean corpuscular hemoglobin; MCHC: Mean corpuscular hemoglobin concentration; MCV: Mean corpuscular volume; RDW: Red cell distribution width.

After recovering from COVID-19, the patient noted many episodes of sinus tachycardia treated with betaloc 25 mg (½ pill/day). She also had episodes of bronchoconstriction and wheezing treated with symbicort (budesonide/formoterol fumarate) 160 (2 times/day) and ventolin (albuterol) when needed. A COVID-19 serology IgG test was done on 2 February 2021 and showed: IgG = 19.58.

On 25 February 2021, and after 3 months already passed since the infection, the patient received the first dose of Pfizer-BioNTech vaccine for COVID-19. After 12 h of receiving the vaccine, she noted fever, chills, myalgia, vertigo, nausea and emesis. Forty-eight hours later, these symptoms resolved completely.

**Figure 5.**
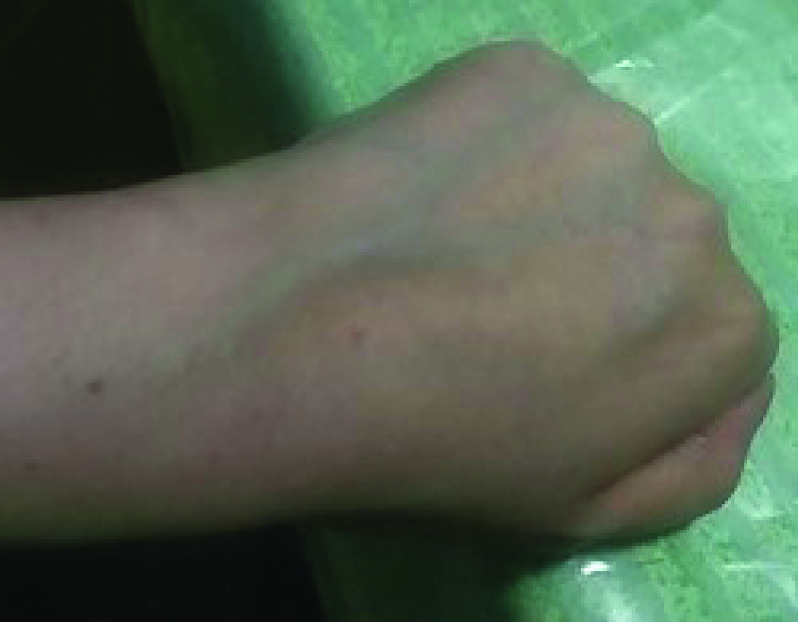
The pattern of evolution of the forearm lesions that occurred following the Pfizer-BioNTech vaccine, progressing from small vesicles into maculopapular lesions, then regressing before they disappear, day 2 postvaccination.

**Figure 6.**
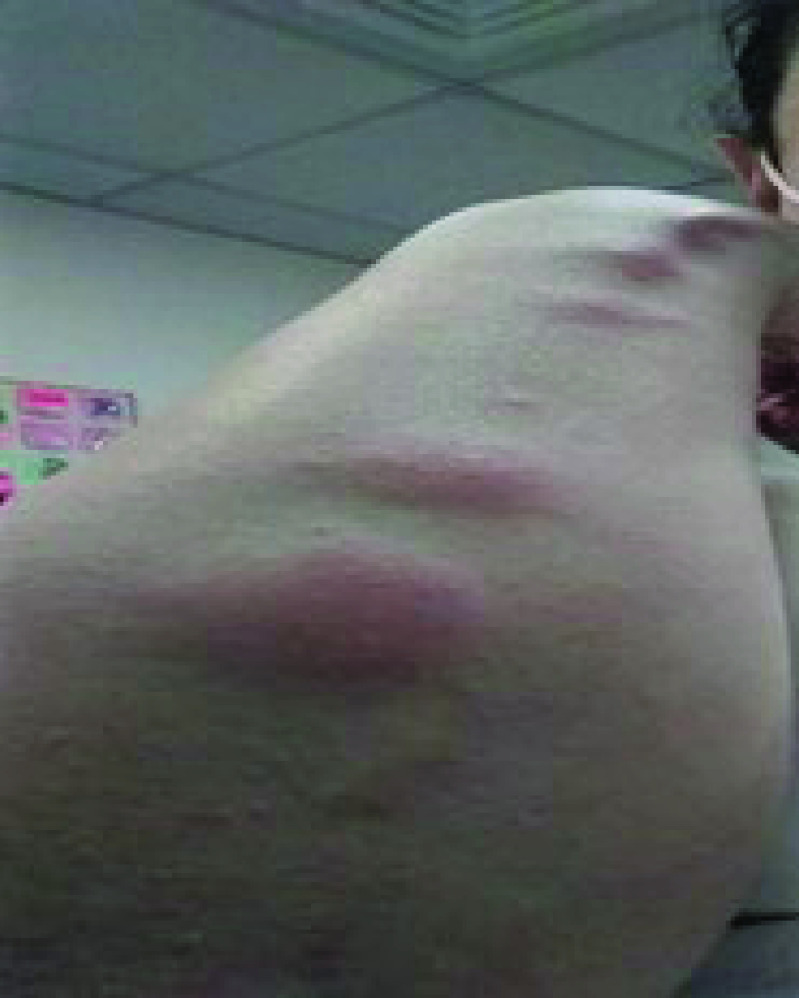
The pattern of evolution of the forearm lesions that occurred following the Pfizer-BioNTech vaccine, progressing from small vesicles into maculopapular lesions, then regressing before they disappear, day 3 postvaccination.

**Figure 7.**
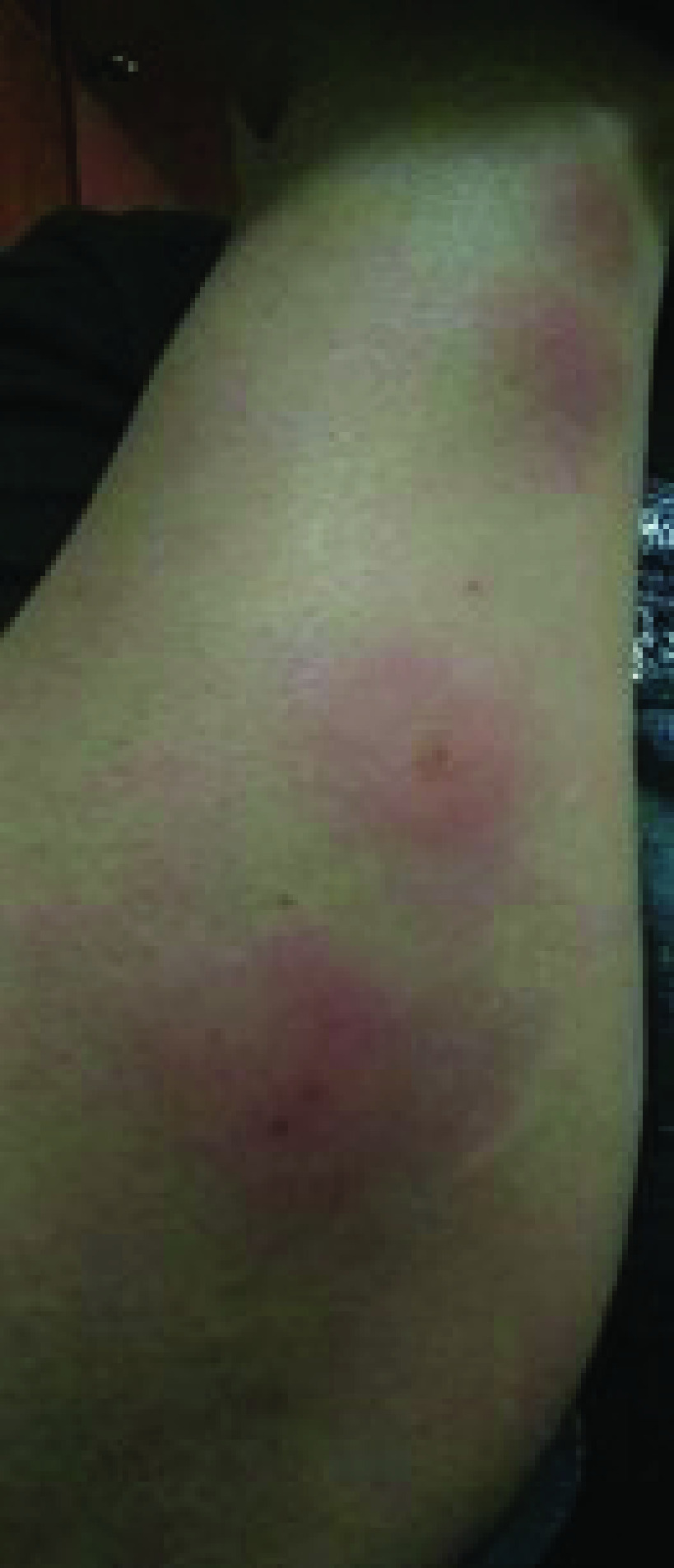
The pattern of evolution of the forearm lesions that occurred following the Pfizer-BioNTech vaccine, progressing from small vesicles into maculopapular lesions, then regressing before they disappear, day 5 postvaccination.

**Figure 8.**
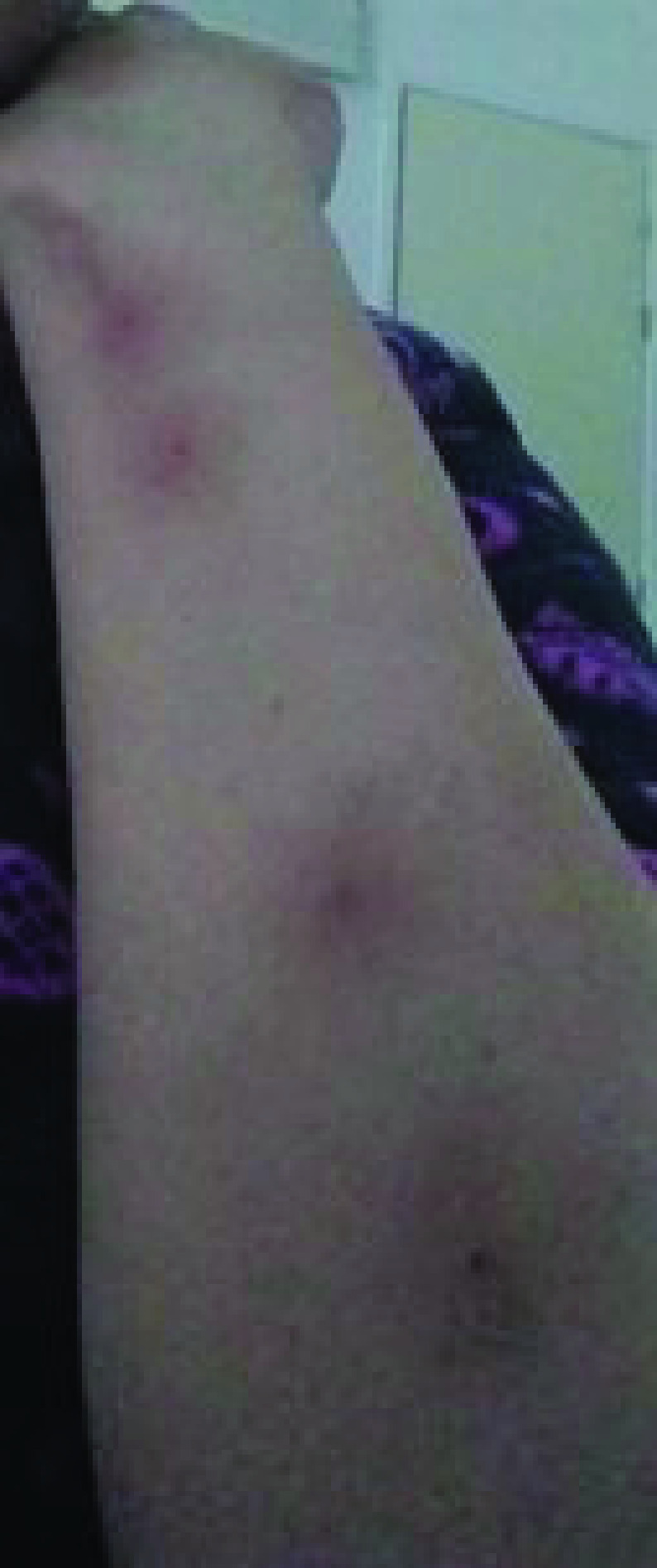
The pattern of evolution of the forearm lesions that occurred following the Pfizer-BioNTech vaccine, progressing from small vesicles into maculopapular lesions, then regressing before they disappear, day 7 postvaccination.

After 24 h of the first dose, an oral ulceration developed, with multiple maculopapular skin lesions, this time on the dorsal part of the hands. These lesions had the same evolution pattern as the skin lesions that appeared during the onset of the COVID-19 infection, and they disappeared quickly. On 28 February 2021, the patient noted four big maculo-papular lesions on the posterior side of the right forearm, these lesions started as small vesicles (Figure 5) and progressed into red, painful, and itchy maculopapular lesions (Figure 6). She presented at the ED on 2 March 2021, with these lesions, as they became swollen. A central vesicle was visible in each lesion. The pattern of evolution of the forearm lesions, progressing from small vesicles into maculopapular lesions, then regressing before they disappear (Figures 5–8).

On day 5 postvaccination (Figure 7), the patient came to the emergency department, we assessed the clinical status of the patient and ordered biological and microbiological investigations. A PCR test was made along with a serology IgG COVID-19 test to assess the immune status of the patient. A sample was taken from the lesions site for microbiological culture. Intravenous corticosteroid therapy was given (Solu-MEDROL 40 mg/12 h), with empiric antibiotic therapy (clindamycin 100 mg/6 h). The patient was admitted for 72-h surveillance at the hospital, during which she had a palpitation episode with a normal EKG. A hypotension episode was noted. No other complications occurred during the surveillance period. The evolution of skin lesions was favorable as they regressed and inflammatory signs disappeared.

**Figure 9.**
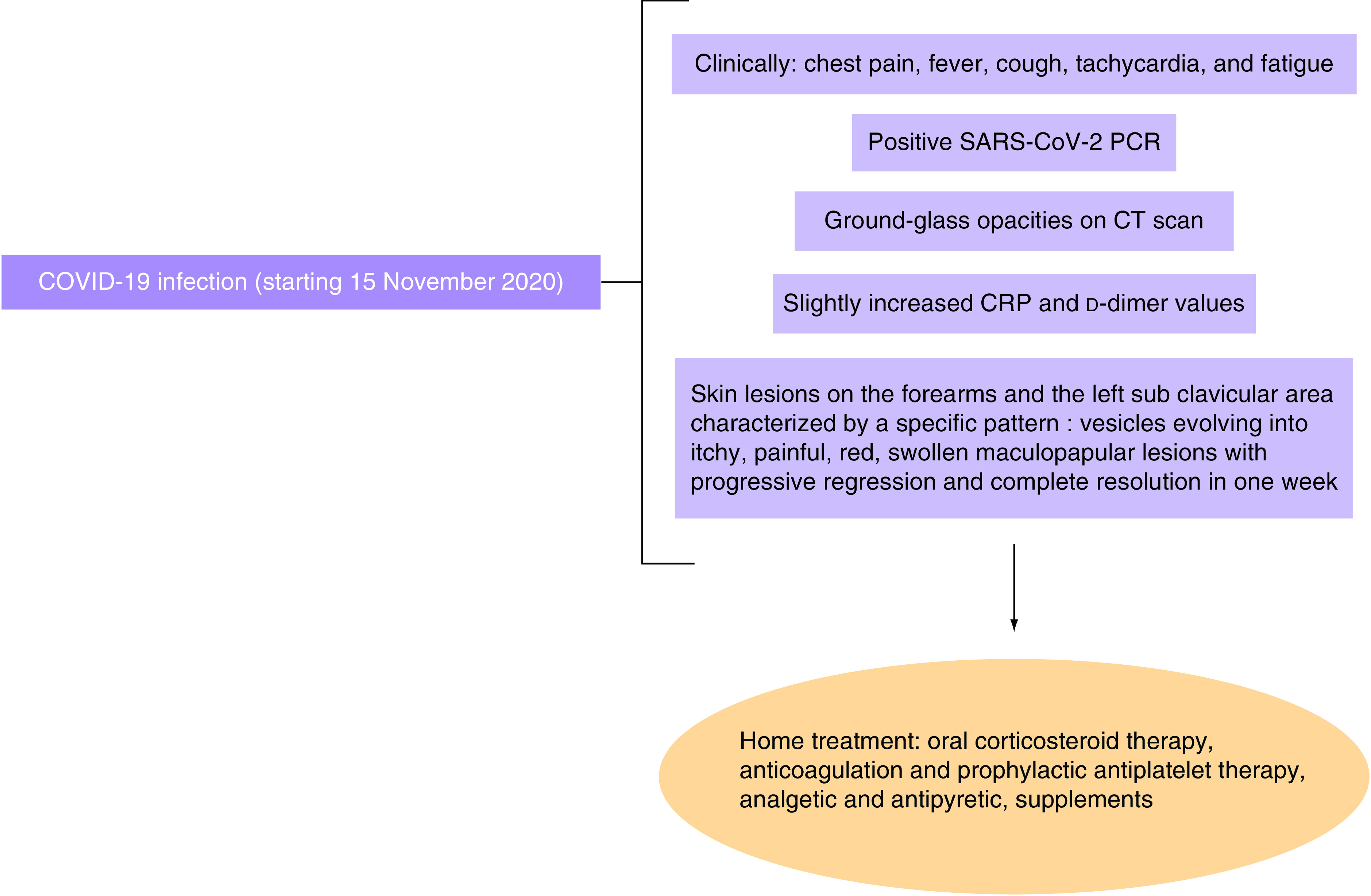
Diagram summarizing patient’s clinical presentation and management during COVID-19 infection.

**Figure 10.**
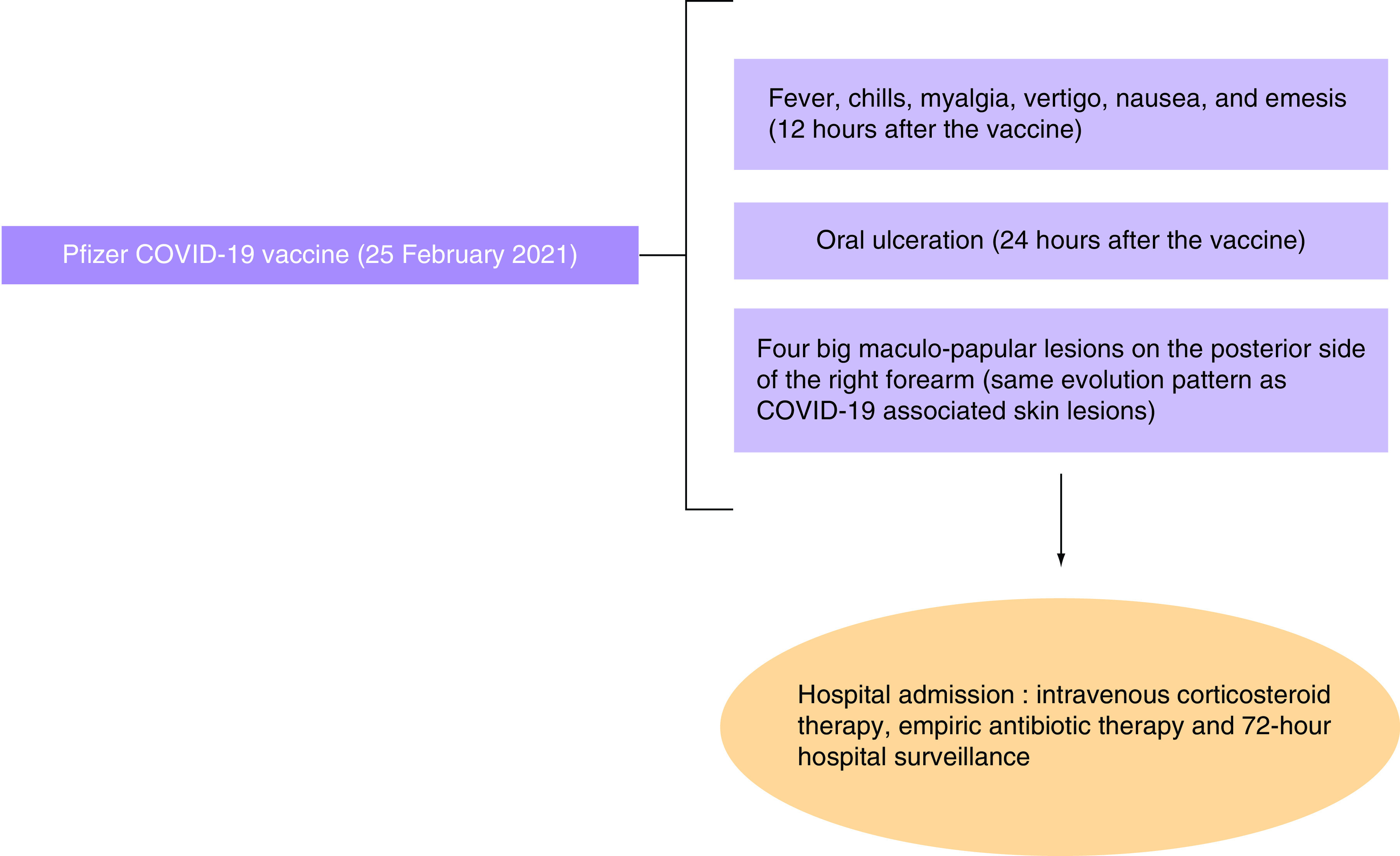
Diagram summarizing patient’s clinical presentation and management after receiving Pfizer-BioNTech COVID-19 vaccine.

Follow-up tests were performed: a SARS-CoV-2 PCR test performed on 27 February 2021 was negative. A serology of anti-SARS-CoV-2 antibodies performed on 2 March 2021 showed a value greater than 100. The lesion's bacterial culture was negative. More tests were done on the following day, showing normal values of complements C3 (128) and C4 (20). CRP and thyroid stimulating hormone (TSH) values were normal. Diagrams summarizing patient’s clinical presentation and management during COVID-19 infection and after COVID-19 vaccination are shown in Figures 9 & 10, respectively.

## Discussion

The physiopathology of COVID-19 infection explains the systemic inflammatory response documented in many studies. This response targets many cellular and molecular sites inducing an end-organ injury. As for the dermatological or cutaneous manifestations, many studies described and noted the presence of this presentation among patients contracting COVID-19 infection [2,4,5]. An itchy, erythematous rash was described as a COVID-19 symptom in a case report of an Italian nurse, and was the first primary care case report of a female patient who developed a skin rash as the first clinical manifestation of COVID-19 [6].

In our case report, the patient presented an atypical pattern of cutaneous lesions starting with small vesicles at day 1, then turning into maculopapular itchy, painful, red, swollen lesions, then regressing progressively with a complete remission at day 7. These findings were described at two specific times: the first time was simultaneously with COVID-19 infection and the second time, day 3 postvaccination with Pfizer-BioNTech COVID-19 vaccine, with a negative PCR test for COVID-19.

The pattern of evolution of the lesions is identical, both after the infection and after the vaccine. The IgG value (>100) 5 days postvaccination shows that the patient had developed a strong and quick immune response against the vaccine, which is typical of the secondary immune response and rules out a re-infection. The primary immune response was developed after the patient contracted the virus, and the IgG value was 19 (test performed on 2 February 2021). The patient immune response to the vaccine is typical of a secondary immune response, which is more quick, intense, and durable than the primary immune response, which the patient had against the virus. Thus, a strong activation of the immune response may have caused the skin lesions to appear. Also, the lesions developed after the vaccine in the right forearm were more intense that those developed during the COVID-19 infection. The lesions were found on both forearms and hands, with a unique lesion on the left sub clavicular area.

IgG SARS-CoV-2 were tested, protective levels were found, and the complements levels were normal. However, the history of the patient having allergy to amoxicillin and history of severe viral infections leads us to some hypotheses:

- Correlation between COVID-19 infection and Pfizer-BioNTech vaccine dermatological reactions
- Maculopapular lesions should be added to the large panoply of COVID-19 infection symptomatology
- Atypical cutaneous lesions pattern should also be considered as a potential COVID-19 symptom

Due to the increase of vaccinations, there has been an increase in mild allergic reactions that can sometimes lead to serious complications and require attention [7]. Therefore, our patient has been admitted to hospital for 72 h, to provide appropriate therapy and surveillance. Main possible complications would be an increase in the intensity of the allergic reaction and bacterial infection of the lesions. The most common signs of delayed-type reactions to vaccines are rashes (i.e., various morphologic forms of maculopapular eruptions) [8]. Our patient had a delayed allergic reaction to the vaccine and presented the most common symptom of this allergic reaction, with other associated symptoms preceding the rash (chills, fever, myalgia).

While there is precedent for the biological plausibility that certain subpopulations become susceptible to vaccine-induced reactions after multiple exposures [9], this may correlate with our patient's case where the repetitive exposure to the COVID-19 antigen (the first being after the infection and the second being after the vaccine) triggered an intense postvaccination allergic reaction. This may also predict a strong allergic reaction if the patient were to receive a second vaccine dose.

It was unlikely that the lesions were due to any kind of medication, noting that the cutaneous manifestations appeared simultaneously with the COVID-19 flu-like symptoms. The patient was not receiving any new medications before the vaccine. A concomitant infection is unlikely, especially that postvaccination CRP value was negative. The pattern similarity after COVID-19 infection and vaccine injection is strong reason to rule out iatrogenic or concomitant infectious causes.

Given the importance of detecting COVID-19 in a global pandemic, it is essential to characterize dermatological manifestations and be aware of unusual lesions pattern that may be correlated or due to COVID-19 infection. It is also important to highlight the correlation between COVID-19 cutaneous manifestations and Pfizer-BioNTech vaccine manifestations for patients with known dermatological reactions in COVID-19. In this case, we used corticosteroid and clindamycin intravenous therapy to reduce the dermatological reaction and we noted a complete remission in 1 week. Concerning the histopathology of COVID-19 related rashes, biopsy results from case studies performed by both Reymundo *et al.* and Mazzitelli *et al.* show perivascular lymphocytic infiltrate in the skin lesions of all patients [10,11].

Since Pfizer-BioNTech vaccine triggers an immune response against the spike protein of the COVID-19 virus, it may be possible to predict some side effects that may be caused by the immune response against the vaccination, among patients who already contracted the virus prior to vaccination. The maculo-papular lesions that occurred in this case report are a type of symptom, but other specific or nonspecific symptoms caused by the COVID-19 infection among some patients may be caused after the vaccine dose injection.

It has been proven that atopy increases the risk of allergic manifestation after COVID-19 vaccine twofold compared with the population, while no correlation with drug allergy has been elucidated [12]. Knowing that the allergic reaction can be extremely painful and intolerable in some cases, a systemic prevention with antihistamines and/or corticosteroids in the absence of contraindications should be considered in some patients with history of severe allergy.

Further studies should be considered to establish clear recommendations on antihistamine and corticosteroid usage for prevention of allergy among patients scheduled to take COVID-19 vaccine, as well as the importance of this prevention and the patients that benefit the most from it.

Other studies are needed to understand the physiopathology of cutaneous manifestation in COVID-19, and to get a clear idea about the risk factors that might be correlated with such dermatological reactions. The relations between patient’s medical history, genetic profile and possible COVID-19 infection manifestations, along with possible allergic and nonallergic side effects of the vaccine, are yet to be elucidated.

Our case study has a limitation: a biopsy of the lesions was not performed, and would have helped further understand immunopathology and histopathology of COVID-19 related rashes and correlate with the histological pattern identified in other studies.

**Summary points**

- Description of a rare side effect of the COVID-19 Pfizer-BioNTech vaccine.
- Description of an uncommon pattern of maculopapular lesions in COVID-19.
- Identification of a pattern of evolution of the skin lesions that is identical during COVID-19 infection and after COVID-19 vaccination.
- Possible correlation between the lesions and immune system abnormalities.

## References

[1] Chen N, Zhou M, Dong X Epidemiological and clinical characteristics of 99 cases of 2019 novel coronavirus pneumonia in Wuhan, China: a descriptive study. Lancet 395(10223), 507–513 (2020). 32007143 10.1016/S0140-6736(20)30211-7 PMC7135076

[2] Matar S, Oulès B, Sohier P Cutaneous manifestations in SARS-CoV-2 infection (COVID-19): a French experience and a systematic review of the literature. J. Eur. Acad. Dermatol. Venereol. 34(11), e686–e689 (2020). 32589293 10.1111/jdv.16775 PMC7361331

[3] Català A, Galván-Casas C, Carretero-Hernández G Maculopapular eruptions associated to COVID-19: a subanalysis of the COVID-Piel study. Dermatol. Ther. 33(6), e14170 (2020). 32779280 10.1111/dth.14170 PMC7436694

[4] Marzano AV, Cassano N, Genovese G, Moltrasio C, Vena GA. Cutaneous manifestations in patients with COVID-19: a preliminary review of an emerging issue. Br. J. Dermatol. 183(3), 431–442 (2020). 32479680 10.1111/bjd.19264 PMC7300648

[5] Gisondi P, PIaserico S, Bordin C, Alaibac M, Girolomoni G, Naldi L. Cutaneous manifestations of SARS-CoV-2 infection: a clinical update. J. Eur. Acad. Dermatol. Venereol. 34(11), 2499–2504 (2020). 32585074 10.1111/jdv.16774 PMC7362144

[6] Serafini A, Kurotschka PK, Bertolani M, Riccomi S. An itchy erythematous papular skin rash as a possible early sign of COVID-19: a case report. J. Med. Case Rep. 14(1), 216 (2020). 33168054 10.1186/s13256-020-02538-y PMC7649709

[7] Chung EH. Vaccine allergies. Clin. Exp. Vaccine. Res. 3(1), 50–57 (2014). 24427763 10.7774/cevr.2014.3.1.50 PMC3890451

[8] Caubet J-C, Ponvert C. Vaccine allergy. Immunol. Allergy Clin. North. Am. 34(3), 597–613 ix (2014). 25017679 10.1016/j.iac.2014.04.004

[9] McNeil MM, DeStefano F. Vaccine-associated hypersensitivity. J. Allergy Clin. Immunol. 141(2), 463–472 (2018). 29413255 10.1016/j.jaci.2017.12.971 PMC6602527

[10] Reymundo A, Fernáldez-Bernáldez A, Reolid A Clinical and histological characterization of late appearance maculopapular eruptions in association with the coronavirus disease 2019. A case series of seven patients. J. Eur. Acad. Dermatol. Venereol. 34(12), e755–e757 (2020). 32495368 10.1111/jdv.16707 PMC7300890

[11] Mazzitelli M, Dastoli S, Mignogna C Histopathology and immunophenotyping of late onset cutaneous manifestations of COVID-19 in elderly patients: Three case reports. World J. Clin. Cases. 9(20), 5744–5751 (2021). 34307634 10.12998/wjcc.v9.i20.5744 PMC8281404

[12] Demir S, Erdenen F, Gelincik A Evaluation of the potential risk factors for drug-induced anaphylaxis in adult patients. Int. Arch. Allergy Immunol. 178(2), 167–176 (2019). 30448840 10.1159/000494130

